# Contribution of intramacrophage stages to *Pseudomonas aeruginosa* infection outcome in zebrafish embryos: insights from *mgtC* and *oprF* mutants

**DOI:** 10.1038/s41598-024-56725-8

**Published:** 2024-03-15

**Authors:** Hélène Hajjar, Laurence Berry, Yongzheng Wu, Lhousseine Touqui, Annette C. Vergunst, Anne-Béatrice Blanc-Potard

**Affiliations:** 1 Laboratory of Pathogens and Host Immunity (LPHI), Université de Montpellier, CNRS-UMR5294, INSERM, Montpellier, France; 2 Institut Pasteur, Université Paris Cité, CNRS UMR3691, Cellular Biology and Microbial Infection Unit, Paris, France; 3 Sorbonne Université, Inserm U938, Centre de Recherche Saint-Antoine (CRSA), Paris, France; 4 Institut Pasteur, Université de Paris Cité, Mucoviscidose et Bronchopathies Chroniques, Paris, France; 5 Bacterial Virulence and Chronic Infections (VBIC), Université de Montpellier, INSERM, U1047 Nîmes, France

**Keywords:** Microbiology, Pathogenesis

## Abstract

*Pseudomonas aeruginosa* often colonizes immunocompromised patients, causing acute and chronic infections. This bacterium can reside transiently inside cultured macrophages, but the contribution of the intramacrophic stage during infection remains unclear. MgtC and OprF have been identified as important bacterial factors when *P. aeruginosa* resides inside cultured macrophages. In this study, we showed that *P. aeruginosa mgtC* and *oprF* mutants, particular the latter one, had attenuated virulence in both mouse and zebrafish animal models of acute infection. To further investigate *P. aeruginosa* pathogenesis in zebrafish at a stage different from acute infection, we monitored bacterial load and visualized fluorescent bacteria in live larvae up to 4 days after infection. Whereas the attenuated phenotype of the *oprF* mutant was associated with a rapid elimination of bacteria, the *mgtC* mutant was able to persist at low level, a feature also observed with the wild-type strain in surviving larvae. Interestingly, these persistent bacteria can be visualized in macrophages of zebrafish. In a short-time infection model using a macrophage cell line, electron microscopy revealed that internalized *P. aeruginosa* wild-type bacteria were either released after macrophage lysis or remained intracellularly, where they were localized in vacuoles or in the cytoplasm. The *mgtC* mutant could also be detected inside macrophages, but without causing cell damage, whereas the *oprF* mutant was almost completely eliminated after phagocytosis, or localized in phagolysosomes. Taken together, our results show that the main role of OprF for intramacrophage survival impacts both acute and persistent infection by this bacterium. On the other hand, MgtC plays a clear role in acute infection but is not essential for bacterial persistence, in relation with the finding that the *mgtC* mutant is not completely eliminated by macrophages.

## Introduction

*Pseudomonas aeruginosa*, a gram-negative bacterium found in soil and water, is one of the most common nosocomial pathogens responsible for acute infections, especially in immunocompromised patients^1^. In patients with Cystic Fibrosis (CF), the viscous mucus layer adhering to the epithelial cells of airway allows *P. aeruginosa* to colonize the CF lungs, where it causes chronic infection that is very difficult to eradicate, increasing the risk of death^1^. Furthermore, increasing antibiotic resistance of *P. aeruginosa* has led the World Health Organization (WHO) to identify this bacterium as a priority in searching for alternative and novel therapeutic strategies^2^. To this aim, appropriate models are required to better understand host–pathogen interactions at the molecular and cellular level, and direct interaction of *P. aeruginosa* with the host immune system.

The zebrafish (*Danio rerio*) is a vertebrate that has demonstrated its importance as a biomedical model to study host–pathogen interaction, particularly with host innate immune cells in the embryo^3^, and it is increasingly used to model human infections^4,5^. The transparency of the embryo offers a unique opportunity to visualize in real time both bacteria and macrophages, which are highly migratory cells capable of the phagocytosis of pathogens, using transgenic zebrafish lines with fluorescent macrophages^6,7^. Zebrafish embryos have been successfully used as a model for *P. aeruginosa* infection to address the role of several bacterial factors, including the type 3 secretion system (T3SS)^8–10^. Different routes of infection have been used, for instance, microinjection of *P. aeruginosa* in a closed compartment causes a localized infection that is particularly suitable to follow in real time the interaction between bacteria and host innate immune cells^11^. Indeed, injection of *P. aeruginosa* in muscle or the hindbrain ventricle has been applied to investigate a role of macrophages in bacterial clearance in vivo during the early phase of acute infection^12^. The failure in clearing *P. aeruginosa* by innate immune cells causes a lethal acute infection in embryos within 24–30 h^8,9^.

Despite being described as an extracellular pathogen, recent data showed that *P. aeruginosa* can reside, at least transiently, inside cultured epithelial cells and macrophages^13–16^. Macrophages play a key role in the immunity of CF lungs^17^. Recent findings have highlighted the pivotal role of *P. aeruginosa* MgtC and OprF in the ability of this bacterium to survive inside host macrophages^12,18,19^. MgtC is an inner membrane protein known to promote intramacrophage growth of several classical intracellular bacteria, including *Salmonella typhimurium*, as well as various non-classical intracellular pathogens^20–22^. OprF is an outer membrane porin that can modulate the production of various virulence factors of *P. aeruginosa*^23,24^. In *P. aeruginosa*, MgtC and OprF are also involved in bacterial escape from the phagosome and cell lysis caused by intracellular bacteria^16^. Upon acute infection in the zebrafish embryo model, both *mgtC* and *oprF* mutant strains of *P. aeruginosa* were significantly attenuated in comparison to the wild-type strain^12,18^. Interestingly, these attenuated phenotypes are dependent on the presence of macrophages, supporting a contribution of both factors in the pathogen-macrophage interaction in vivo^12,18^.

In the present study, we first performed a comparative analysis of the virulence of the wild-type *P. aeruginosa* strain PAO1, as well as *mgtC* and *oprF* mutants, in mouse and zebrafish acute infection models. Then, we investigated for the first time the behavior of these strains at a stage different from acute infection in zebrafish embryo to address bacterial persistence and interaction with host macrophages. To extend our in vivo findings, we investigated in detail the intracellular localization of these three strains in infected cultured rodent macrophages using electron microscopy.

## Material and methods

### Bacterial strains and growth conditions

Bacterial strains and plasmids used in this study are described in Table 1. *P. aeruginosa* strain PAO1, and PAO1-derived *oprF* and *mgtC* mutants express GFP constitutively by the introduction of plasmid pMF230 (Addgene).

**Table 1:** Bacterial strains used in the study.

Name	Description	Reference
PAO1	Wild-type	Laboratory collection
H636	Δ*oprF*	^35^
PAO1 Δ*mgtC*	Δ*mgtC*	^18^
PAO1 pMF230	Wild-type GFP*mut2*, Amp^r^	^16^
H636 pMF230	Δ*oprF* GFP*mut2*, Amp^r^	^16^
PAO1 Δ*mgtC* pMF230	Δ*mgtC* GFP*mut2*, Amp^r^	^16^

### In vivo experiments in mice

Adult C57BL/6J were purchased from Charles River Laboratories (France) and housed in the Institut Pasteur animal facilities. All experiments were performed in compliance with the French and European regulations on care and protection of laboratory animals (EU Directive 2010/63, French Law 2013-118, February 6th, 2013). The studies were approved by the Ethics Committee of Institut Pasteur (reference 2014-0014) with the infection protocol 04.146. Mice were infected intranasally as described previously^25^. In brief, mice were anesthetized by intraperitoneal injection of ketamine (Imalgene 1000^®^, 40 mg/kg)/xylazine (Rompun^®^, 8 mg/kg) suspended in PBS. The intranasal infection was conducted in the anesthetized animals (6 mice per group) with *P. aeruginosa* PAO1, PAO1∆*mgtC* or PAO1∆*oprF* (1.5 × 10^7^ CFU) suspended in 50 μl of PBS. The animal mortality was monitored twice daily up to 60 h.

### Ethics statement for zebrafish

Animal experimentation procedures were conducted at the University of Montpellier by following the 3Rs—Replacement, Reduction and Refinement principles according to the European Union guidelines for handling of laboratory animals (http://ec.europa.eu/environment/chemicals/lab_animals/home_en.htm) and were approved by the Direction Sanitaire et Vétérinaire de l'Hérault and Comité d’Ethique pour l’Expérimentation Animale under reference CEEA-LR-B4-172-37 and APAFIS #36309-2022040114222432 V2. Larvae were treated with the anesthetic Tricaine (400 µg/ml) before bleaching. The study is reported in accordance with ARRIVE guidelines.

### Zebrafish lines, husbandry and maintenance

Wild-type AB zebrafish (ZIRC Cat# ZL1, RRID:ZIRC_ZL1) or *tg(mfap4: mCherry-F)* transgenic fish with macrophages displaying red fluorescence^26^ were maintained at 28 °C under standard conditions^27^. Adult fish were reared in accordance with international guidelines set out in EU Animal Protection Directive 2010/63/EU. Eggs were obtained by natural spawning, collected in petri dishes and incubated at 28 °C in water with 60 μg/ml ocean salts supplemented with 0.1% methylene blue. For experiments, larvae were staged at 1-day post-fertilization (dpf) and used from 2 to 6 dpf.

### Microinjection of *P. aeruginosa* into zebrafish embryos

The bacteria were freshly streaked out on LB-Agar plates from glycerol stocks kept at − 80 °C. Bacterial strains were grown in LB medium to mid-log phase (OD_600_ = 0.7 to 0.8), recovered by centrifugation and washed twice in Phosphate-Buffered Saline (PBS). A 26-gauge needle was used to homogenize suspensions that were diluted in PBS at about 10^9^ bacteria/ml. Phenol red (0.1% final) was added to facilitate visualization of the injection process.

Infection was carried out by the direct microinjection of 1.5 nl of bacterial suspension, containing 2000–2500 Colony Forming Unit (CFU), into the hindbrain ventricle of previously dechorionated embryos anesthetized with 400 µg/ml Tricaine (from 4 mg/ml stock, Sigma, France) at 50 h post-fertilization (hpf). To follow infection kinetics, larvae were individually transferred into a multi-well plate and incubated using a light/dark cycle at 28 °C with the melanization inhibitor Phenylthiourea (PTU) 1X. The number of dead embryos was counted based on the absence of heartbeat after visual inspection to determine survival curves.

### Bacterial loads (CFU) in infected embryos

Groups of five infected larvae were collected at 24, 48, 72 and 96 h post-infection (hpi). The experiments were performed in triplicate. The larvae were lysed individually using a pestle in 100 µl of PBS. Then 100 µl of Triton X-100 was added to rinse off all residual bacteria from the pestle (PBS-Triton 1%). After a period of 10 min to allow Triton to fully lyse cells and to release intracellular bacteria, 100 µl was plated on LB plates containing 100 μg/ml ampicillin. Several dilutions were plated when needed, usually for the 24 hpi time-point. Only fluorescent colonies were considered for CFU counts and are represented as individual values in scatter dot plots, prepared with GraphPad Prism 8.0.2 Software (https://www.graphpad.com/updates/prism-802-release-notes). A black horizontal line indicates the mean. An aliquot (20 µl) of the well medium was also plated and fluorescent colonies were counted to assess the presence of *P. aeruginosa* in the medium. At 48, 72 and 96 hpi, if 20 or more fluorescent colonies were found, which occurred at low frequency (< 10%) and only with PAO1 and Δ*mgtC* strains, the corresponding zebrafish was not included in the analysis. Plasmid pMF230 is stable for all strains in non-selective medium in vitro upon serial dilution passages. We can, however, not exclude a low percentage of plasmid loss within the host, which might result in an underestimation of CFU numbers at later infection times.

### Live imaging of zebrafish larvae

After anesthesia with tricaine (400 µg/ml), larvae were mounted in 1% low melting point agarose (Sigma, france) in 35 mm glass-bottom dishes (FluoroDish, World Precision Instruments, UK). Alive zebrafish larvae were imaged using the ANDOR CSU-W1 confocal spinning disk on an inverted NIKON microscope (Ti Eclipse) with ANDOR Neo sCMOS camera (20× air/NA 0.75 objective) at different time-points post injection, up to 96 hpi. The excitation wavelength of the green laser (for the bacteria expressing GFP) was 488 nm, the emission wavelength 525 and the exposure time was 0.15 s. The excitation wavelength of the red laser (for the macrophages expressing mCherry) was 561 nm, the emission wavelength 607 and the exposure time was 0.5 s. The exposure time for the white light (DIC) was 0.1 s. Z-stack images of approximately 100 planes with 1 µm intervals were acquired at 28 °C to study the localization of bacteria (inside or outside the host macrophages) and the presence of bacterial clusters. 3D files were generated from z-stack image acquisition with the iQ3 .6.5 software (Andor; https://andor.oxinst.cn/products/iq-live-cell-imaging-software/andor-iq3). Images in the manuscript were prepared using Imaris 9.6 (Andor Technology, https://imaris.oxinst.com/versions/9-6), after conversion of the files using Imaris file converter. Image properties were set at x = 0.319, y = 0.319, z = 1 to set the scale and channels set to the correct colour. Stacks, individual planes and orthogonal views are shown as indicated in the figure legends. Image intensities were only adapted for Fig. 3B green (reduced), Fig. 4A red (increased) and 4B (reduced green and red background). Additional artistic 3D representations are shown for some of the images (settings: Surfaces, background subtraction, diameter of largest sphere: 20 µm for macrophages, 1 µm for bacteria).

### Transmission electron microscopy (TEM)

J774A.1 cells were kept at 37 °C in 5% CO_2_ in Dulbecco’s modified Eagle medium (DMEM) (Gibco) supplemented with 10% fetal bovine serum (FBS) (Gibco). *P. aeruginosa* infection of J774 macrophages on glass coverslips was completed as previously described^18^. For preparation of samples for TEM analysis, infected cells that have been treated after phagocytosis for 2.5 h with gentamicin (to eliminate extracellular bacteria) were fixed with 2.5% glutaraldehyde. Acetonitrile series were used for dehydration. Epon 118: acetonitrile 50:50, followed by two times for 1 h in 100% epon impregnated samples as described previously^16^. A Leica UC7 ultramicrotome (Leica microsystems) was utilized to cut ultrathin sections of 70 nm. Those sections were then counterstained with uranyl acetate and lead citrate and imaged with a Jeol 1200 EXII transmission electron microscope. All chemicals were from Electron Microscopy Sciences (USA) and solvents were from Sigma. Images were treated using Fiji software (https://imagej.net/software/fiji/).

### Statistical analysis

Graphs and data analysis were done using GraphPad Prism 8.0.2 (https://www.graphpad.com/updates/prism-802-release-notes) software. The specific statistical tests used to evaluate the significant differences between groups is indicated in the figure legends. Kaplan–Meier survival analysis and log-rank (Mantel-Cox) test were utilized for survival curves whereas ordinary One-way-ANOVA followed by Sidak’s test was used for CFU. Statistically significant included p-values are lower than 0.05. *P < 0.01, **P < 0.001, ***P < 0.0001. The p-values and the number of independent experiments (biological replicates) are indicated in the figure legends.

## Results

### MgtC and OprF are key virulence factors of *P. aeruginosa* in mammalian and non-mammalian animal models

**Figure 1:**
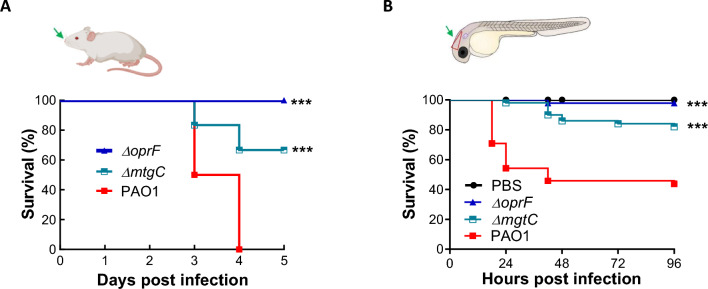
Host survival following *P. aeruginosa* infection in mice and zebrafish. (**A**) *P. aeruginosa* is administrated intranasally in mice (created with BioRender.com). Mice were infected with 1.5 × 10^7^ CFU of *P. aeruginosa* PAO1, PAO1∆*mgtC* or PAO1∆*oprF* suspended in 50 µl of Phosphate Buffer Saline (PBS)*.* Mortality was recorded twice a day until 60 h, n = 6 animals per group, representative of 2 independent experiments. (**B**) *P. aeruginosa* is microinjected into the hindbrain ventricle of zebrafish larvae. 2 days post-fertilization (dpf) AB zebrafish larvae were microinjected with 1.5 nl of PBS (control) or between 2000 and 2800 CFU of *P. aeruginosa* PAO1, PAO1∆*mgtC* or PAO1∆*oprF*. Mortality was recorded at 18, 24, 42, 48, 72 and 96 h post-injection (hpi). Data are representative of 3–4 pooled, independent experiments. Pooled numbers of individual fish are as follows: n = 22 (PBS), 48 (PAO1), 47 (PAO1Δ*oprF*) and 50 (PAO1Δ*mgtC*). A Kaplan–Meier survival analysis and log-rank (Mantel-Cox) test demonstrated a significant reduction in survival in PAO1 compared to both mutants for both animal models (mouse and zebrafish). ***p < 0.001.

Since the virulence of m*gtC* and *oprF* mutant strains, both in a PAO1 background, has never been tested in a mammalian model, our first objective here was to analyze virulence of both mutants in mice using intranasal administration of bacteria to induce an acute pulmonary infection. Both *mgtC* and *oprF* mutants exhibited significantly attenuated virulence compared to the PAO1 WT strain as established by survival curves, with the *oprF* mutant unable to cause death until 5 days of infection (Fig. 1A). These data are consistent with previous results obtained after intravenous (iv) injection in a zebrafish embryo infection model^12,18^, confirming that MgtC and OprF are key virulence factor of *P. aeruginosa*, and that the virulence profiles of the three *P. aeruginosa* strains are similar in mice and zebrafish models.

In order to study bacterial behavior at the cellular level in vivo, we chose to inject bacteria in a localized compartment, the hindbrain ventricle (HBV) of 2 dpf zebrafish embryos, which is very suitable for analysis of the interaction of bacteria with macrophages^12^. Therefore, we first analyzed the infection pattern of the wild-type and the two mutants in this localized infection model. The *mgtC* and *oprF* mutants showed attenuated virulence compared to the PAO1 WT strain (Fig. 1B), with the *oprF* mutant being the least virulent, validating the use of the zebrafish embryo to address *P. aeruginosa* pathogenesis using the HBV infection model.

### Analysis of bacterial load in zebrafish larvae up to 4 days post-infection

**Figure 2:**
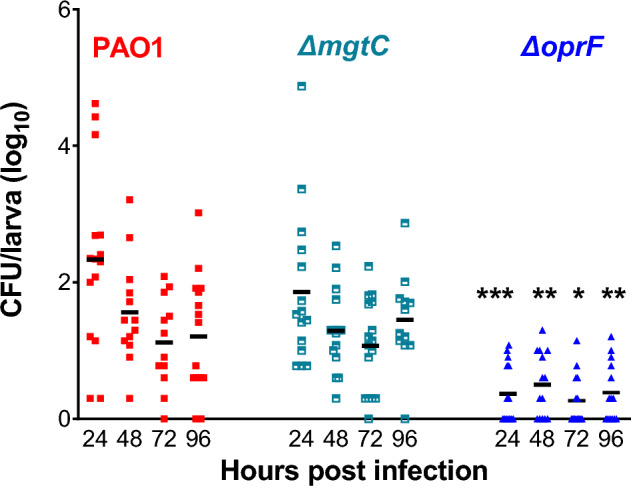
Bacterial loads in infected zebrafish larvae. Quantification of bacterial loads at different times post infection by counting Colony Forming Units (CFU) from AB larva injected with approximately 2500 bacteria of PAO1 WT strain, and *mgtC* and *oprF* mutants. CFU were counted only in live larvae in 3 independent experiments. Each symbol represents an individual larva and black horizontal lines indicate the median values. Statistically significant lower CFU were counted with *oprF* mutant, but not *mgtC* mutant, compared to PAO1 WT, indicating that Δ*oprF* bacteria are mostly eliminated*,* whereas PAO1 and Δ*mgtC* bacteria can persist. *p < 0.05, **p < 0.01, ***p < 0.001 (Ordinary One-Way ANOVA followed by Sidak’s test)*.*

In zebrafish, about 50% of embryos injected with the wild-type PAO1 strain died within the first 24 h after infection, which is considered as an acute infection, while embryos that survived 2 days after infection did not die within the time frame of the experiment (Fig. 1B). To assess whether embryos that were able to survive infection with PAO1 for 4 days had cleared the bacteria or not, we determined bacterial CFUs in live zebrafish larvae at different time-points post-infection (24, 48, 72 and 96 hpi). Clearly, at 24 hpi most live larvae contained fewer bacteria than the initial inoculum of 2000 CFU, indicating host killing of bacteria after injection. Larvae, surviving up to the end point of the assay (96 hpi, Fig. 2), had stable low bacterial burden after 48 hpi, indicating bacterial persistence in surviving embryos. Although more larvae died after injection with wild-type PAO1 than with the *mgtC* mutant, unexpectedly, both strains exhibited similar CFU profiles with between 15 and 30 CFU (median values) in surviving larvae between 2 and 4 dpi (Fig. 2). The few larvae that contained large numbers of PAO1 or the *mgtC* mutant bacteria at 24 hpi (> 1000) most likely are larvae that would have died from acute infection. In contrast, surviving larvae infected with the *oprF* mutant had mostly cleared the bacteria, and only some had very few bacteria (mean of 2–5 bacteria per embryo) (Fig. 2).

Taken together, PAO1 causes an acute lethal infection in about 50% of the larvae, while surviving larvae develop a persistent infection with relatively low bacterial burden, with reproducible variation in CFU between larvae. The results demonstrate that OprF plays an important role in bacterial persistence and induction of acute infection, while MgtC seems not essential for persistence, yet has an important contribution to acute fatal infection.

### Real time imaging of persistent *P. aeruginosa* WT and *mgtC* mutant in zebrafish larvae

**Figure 3:**
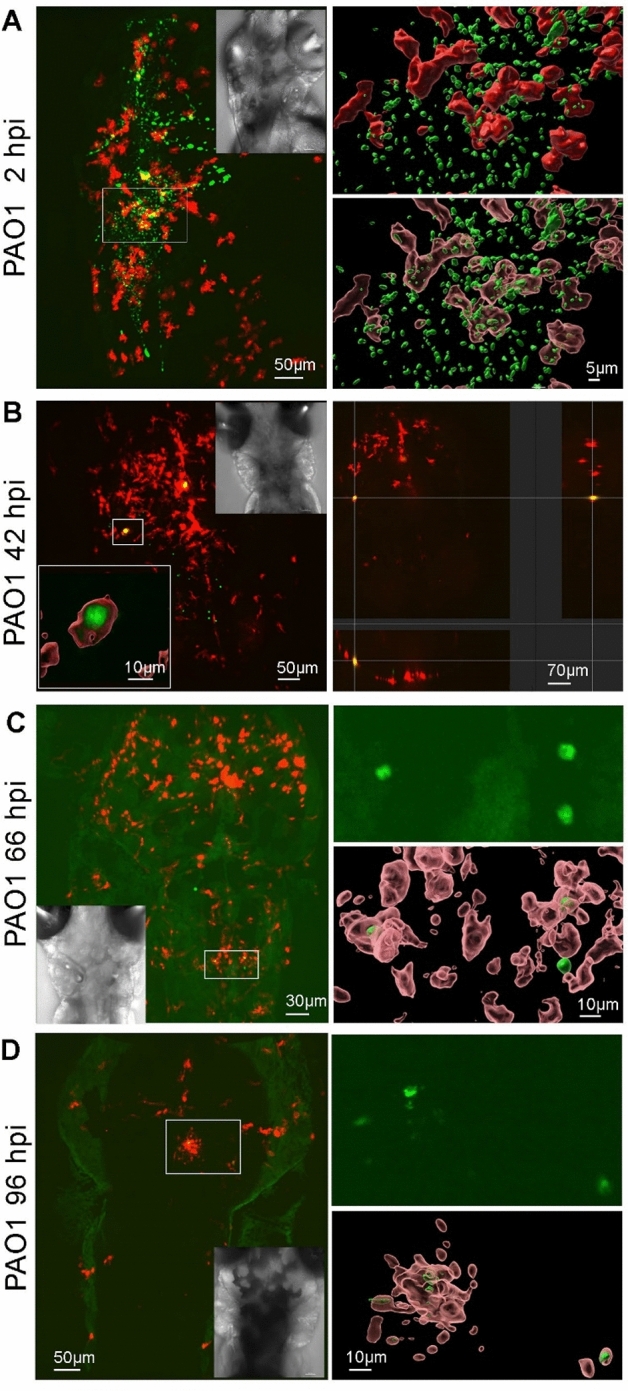
Live imaging of *P. aeruginosa* PAO1 WT in *tg(mfap4:mCherry-F)* zebrafish larvae. Representative images of *tg(mfap4:mCherry-F)* larvae infected with approximately 2500 CFU of PAO1-GFP in the HBV at 2 dpf (days post-fertilization). Live larvae were imaged in real-time with a confocal spinning microscope at different time-points post-injection: 2 hpi (**A**), 42 hpi (**B**), 66 hpi (**C**), and 96 hpi (**D**). Left panels: Z-stack of original images (114, 186, 137 and 50, respectively) showing overlay of green (bacteria expressing GFP) and red (macrophages expressing mCherry) channels. The insets show the bright field images (**A**–**D**) and 3D surface reconstruction of a bacterial cluster in a macrophage (left-bottom in **B**). Right panels: 3D reconstruction of the indicated area on the left showing closed and open surfaces to accentuate intramacrophage bacteria (**A**), orthogonal view showing intramacrophage bacteria (**B**), untreated green filter image and open surface reconstruction (**C**,**D**).

We then aimed to visualize persisting bacteria in embryos between 2 to 4 dpi, specifically the interaction of these bacteria with host macrophages. Therefore, bacterial strains carrying a reporter plasmid that constitutively expresses GFP were injected into the HBV of 2 dpf embryos from the transgenic fish line *tg(mfap4:mCherry-F)* in which macrophages are labelled with a red fluorescent protein. Real-time, non-invasive live imaging was conducted using a confocal spinning disk microscope. Imaging of embryos that had been injected with the wild-type strain confirmed the initial high bacterial load and presence in macrophages at 2 hpi (Fig. 3A), as reported earlier^12^. We then imaged the HBV area of individual live embryos injected with the wild-type strain at 2, 3 or 4 dpi and assessed the maximal projection of z-stacks and 3D reconstruction (Fig. 3B–D). In agreement with CFU data, fluorescent bacteria could be seen up to 4 dpi in surviving larvae. Such persisting bacteria sometimes appeared as small clusters (Fig. 3B). Importantly, persisting *P. aeruginosa* PAO1 WT bacteria were detected inside macrophages, as visualized in orthogonal view (Fig. 3B) and by 3D reconstruction (Fig. 3B–D). Small bacterial clusters were also seen outside macrophages, although we could not determine whether these were present in other cell types (Fig. 3C), or in potential vesicles issued from macrophages (Fig. 3D).

**Figure 4:**
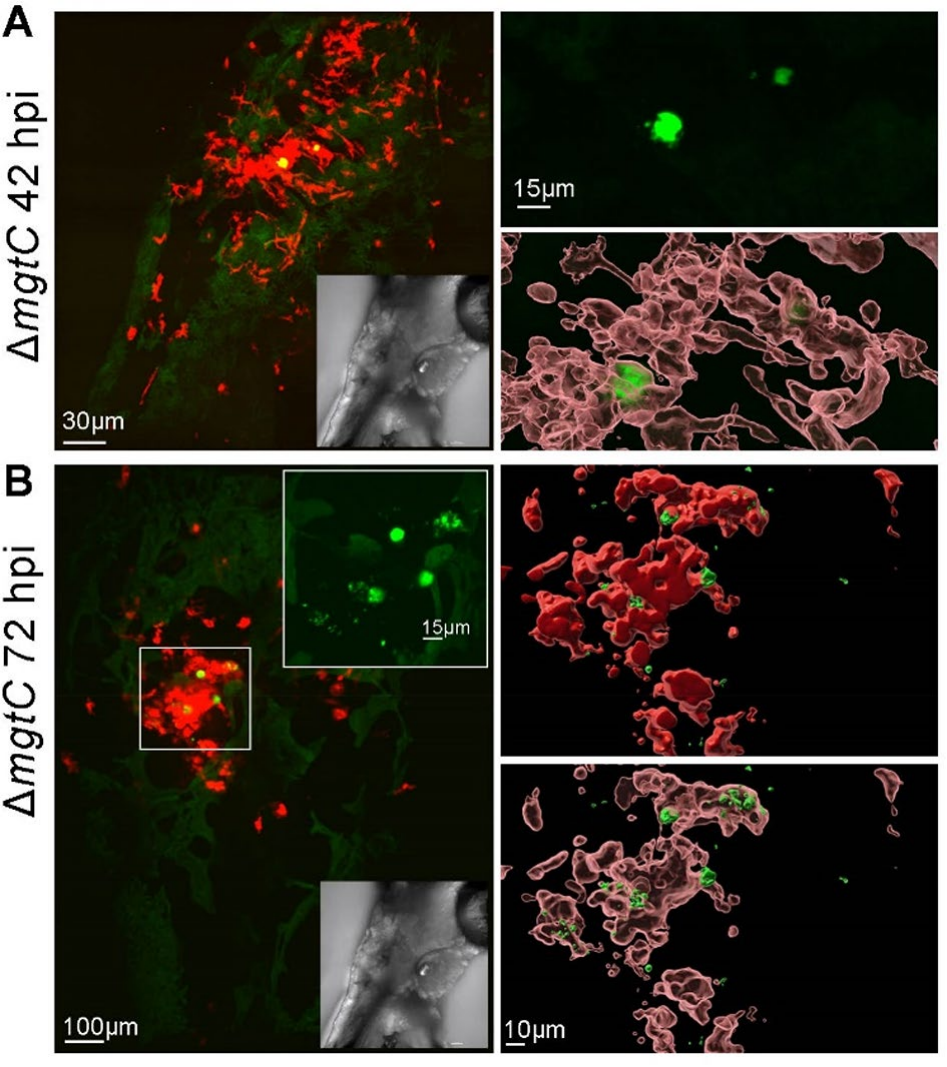
Live imaging of *Pseudomonas aeruginosa* PAO1*ΔmgtC* in *tg(mfap4:mCherry-F)* zebrafish larvae. Representative images of *tg(mfap4:mCherry-F)* larvae infected with approximately 2500 CFU of PAO1 Δ*mgtC*-GFP in the HBV at 2 dpf. Live larvae were imaged in real-time with a confocal spinning microscope, shown at 42 hpi (**A**) and 72 hpi (**B**). Left panels: stack of images (109 and 70 respectively) of green (bacteria expressing GFP) and red (macrophages) channels. The insets show the bright field images (**A**,**B**). In (**B**), the stack of images with the green filter is shown as inset. Right panels: 3D reconstruction of the indicated area on the left using the raw image stack with the green filter (top right panel **A**) and its overlay in open surface projection showing macrophages; open and closed surface structures visualizing both intramacrophage and extra-macrophage bacteria (**B**).

**Figure 5:**
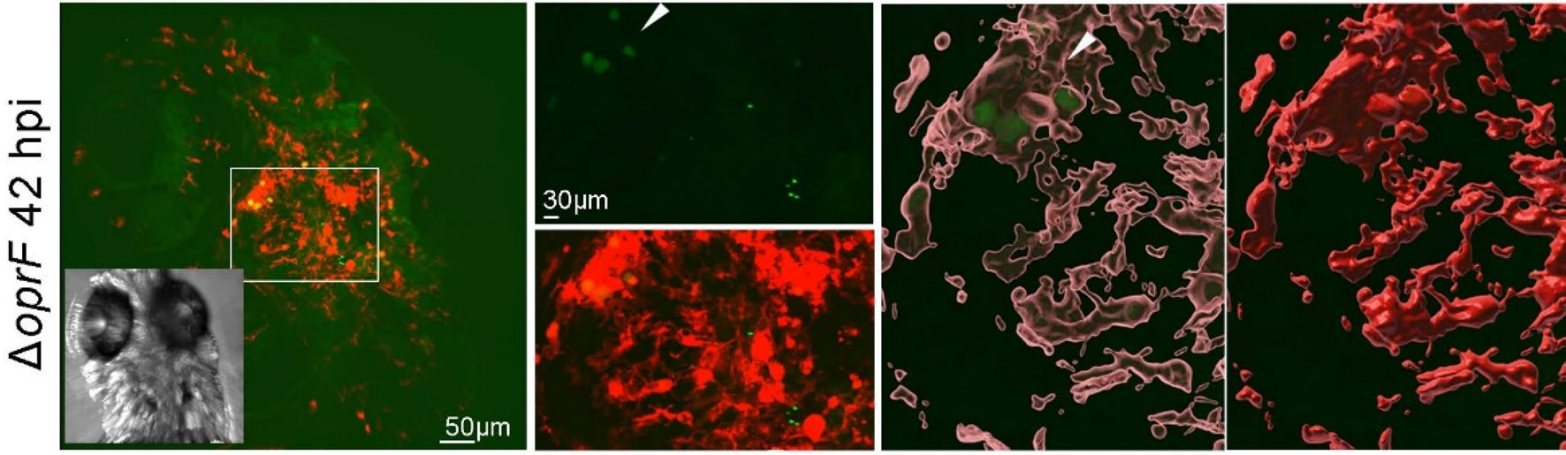
Live imaging of *Pseudomonas aeruginosa* PAO1*ΔoprF* in *tg (mfap4:mCherry-F)* zebrafish larvae. Representative image of *tg(mfap4:mCherry-F)* larvae infected with approximately 2500 CFU of PAO1 Δ*oprF*-GFP in the HBV at 2 dpf imaged in real-time with a confocal spinning microscope at 42 hpi. Left panel: original stacked images (124) showing overlay of green (bacteria expressing GFP) and red (macrophages expressing mCherry) channels. The insert shows the bright field image. The indicated area is shown enlarged on the right, top: green filter only, showing diffuse GFP signal (arrow head). Two images on the right: 3D reconstruction with open and solid surfaces for the red channel using the original (not surface projection) green signal to visualize the intramacrophage localisation of the diffuse signal.

Real time imaging of zebrafish larvae injected with the *mgtC* mutant from 2 dpi until 4 dpi showed, similar to the wild-type strain and consistent with CFU data (Fig. 2), that bacteria can be visualized at later time points in infection, either individually or clustered (Fig. 4). Clustered bacteria could be detected inside macrophages, as shown by 3D reconstruction (Fig. 4A,B). In embryos infected with the *oprF* mutant, bacteria were difficult to detect, which is in line with the low CFU count per larvae (Fig. 2). Occasionally, persisting bacterial clusters were visualized inside macrophages, as shown by 3D reconstruction, some of which had diffuse fluorescence, suggesting that bacteria had been degraded in macrophages (Fig. 5).

Taken together, real time imaging of infected embryos supports the ability of *P. aeruginosa* wild-type and *mgtC* mutant bacteria, but not the *oprF* mutant, to persist at low level in zebrafish embryos up to at least 3–4 dpi, which agrees with the CFU quantification. Moreover, the imaging showed that the wild-type strain and *mgtC* mutant were able to reside inside macrophages at late time points. Due to low numbers of bacteria, it was however not feasible to quantitate precise numbers of intra/extramacrophage bacteria based on live imaging, which is a limitation to determine the contribution of intracellular bacteria in persistence.

### Fate of *P. aeruginosa* strains upon infection of cultured macrophages

**Figure 6:**
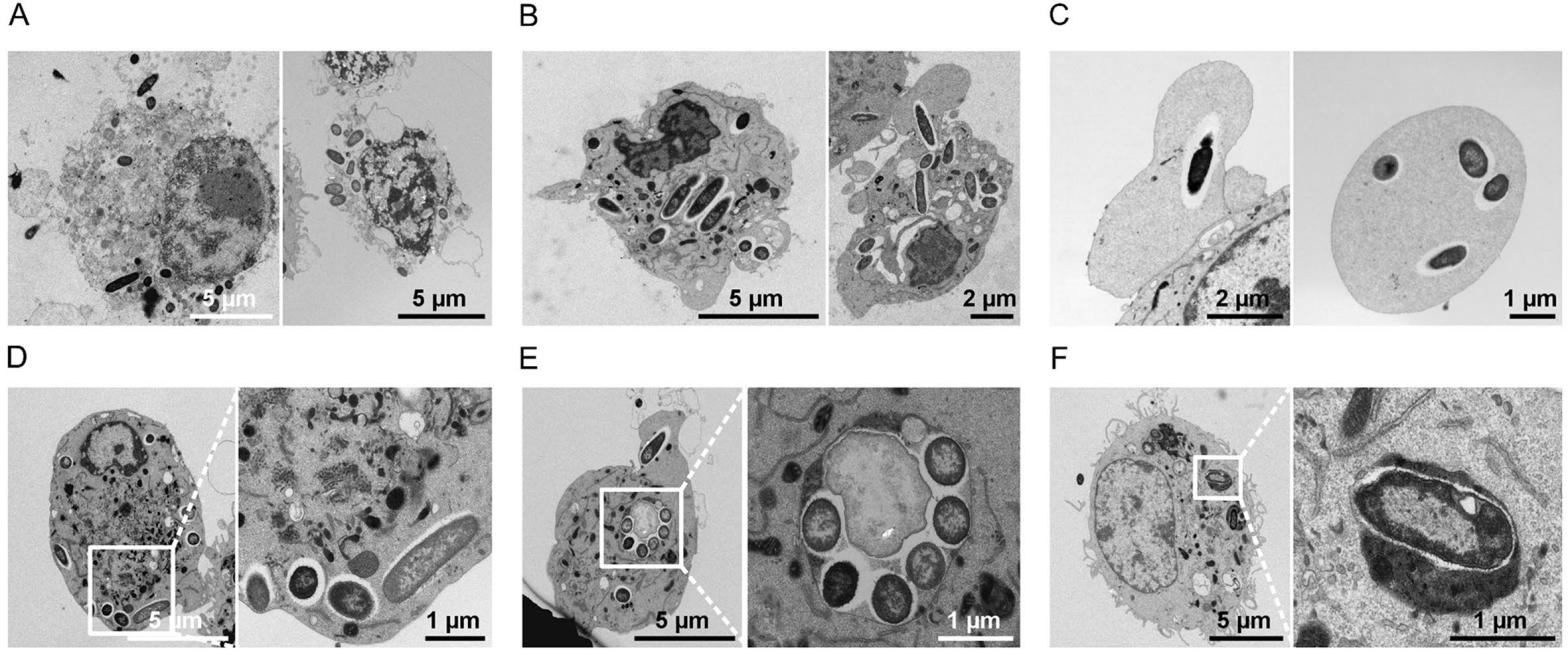
TEM analysis of cultured macrophages infected with PAO1 wild-type strain. J774 macrophages were infected with *P. aeruginosa* PAO1 for 2.5 h before fixation for TEM analysis. Macrophages could be observed in bad conditions i.e., either lysed with released bacteria (**A**) or dying with bacteria in the cytoplasm (**B**). Vesicles issued from macrophages can harbor bacteria (**C**). Phagocytized bacteria can also reside in healthy macrophages, either in the cytoplasm (**D**), inside a vacuole (**E**) or rarely inside a phagolysosome (**F**).

We have previously used macrophage cell line J774 to investigate the fate of intracellular *P. aeruginosa* after phagocytosis and gentamicin treatment to remove extracellular bacteria, and demonstrated cell lysis caused by intracellular bacteria^16^. A quantitative analysis using fluorescent wild-type strain, *mgtC* or *oprF* mutants showed that both MgtC and OprF were involved in the ability of intracellular *P. aeruginosa* to escape from the phagosome and lyse macrophages in culture^16^. Using transmission electron microscopy (TEM), we have previously shown that *P. aeruginos*a PAO1 WT first resided in phagosomal vacuoles at early time point after phagocytosis (25 min) and subsequently could be detected in the cytoplasm at 2.5 h after phagocytosis^16^. In the present study, we extended the qualitative TEM analysis of J774 macrophages infected with the wild-type strain, *mgtC* or *oprF* mutants at 2.5 h after phagocytosis. Imaging of PAO1-infected cells showed different outcomes upon internalization by macrophages. We could visualize lysed macrophages that released intracellular bacteria (Fig. 6A). Bacteria could also be found in the cytoplasm of damaged macrophages (Fig. 6B) or in blebs (Fig. 6C). On the other hand, bacteria were also detected to reside in seemingly healthy macrophages, either in the cytoplasm (Fig. 6D) or in vacuoles (Fig. 6E). Non-damaged infected cells with bacteria found in vacuoles filled with heterogeneous electron dense material were also rarely observed (Fig. 6F), suggesting bacterial degradation by phago-lysosomal fusion. Infection with wild-type PAO1 thus showed diverse patterns, from bacterial release by lysed macrophages to bacterial elimination by macrophages.

**Figure 7:**
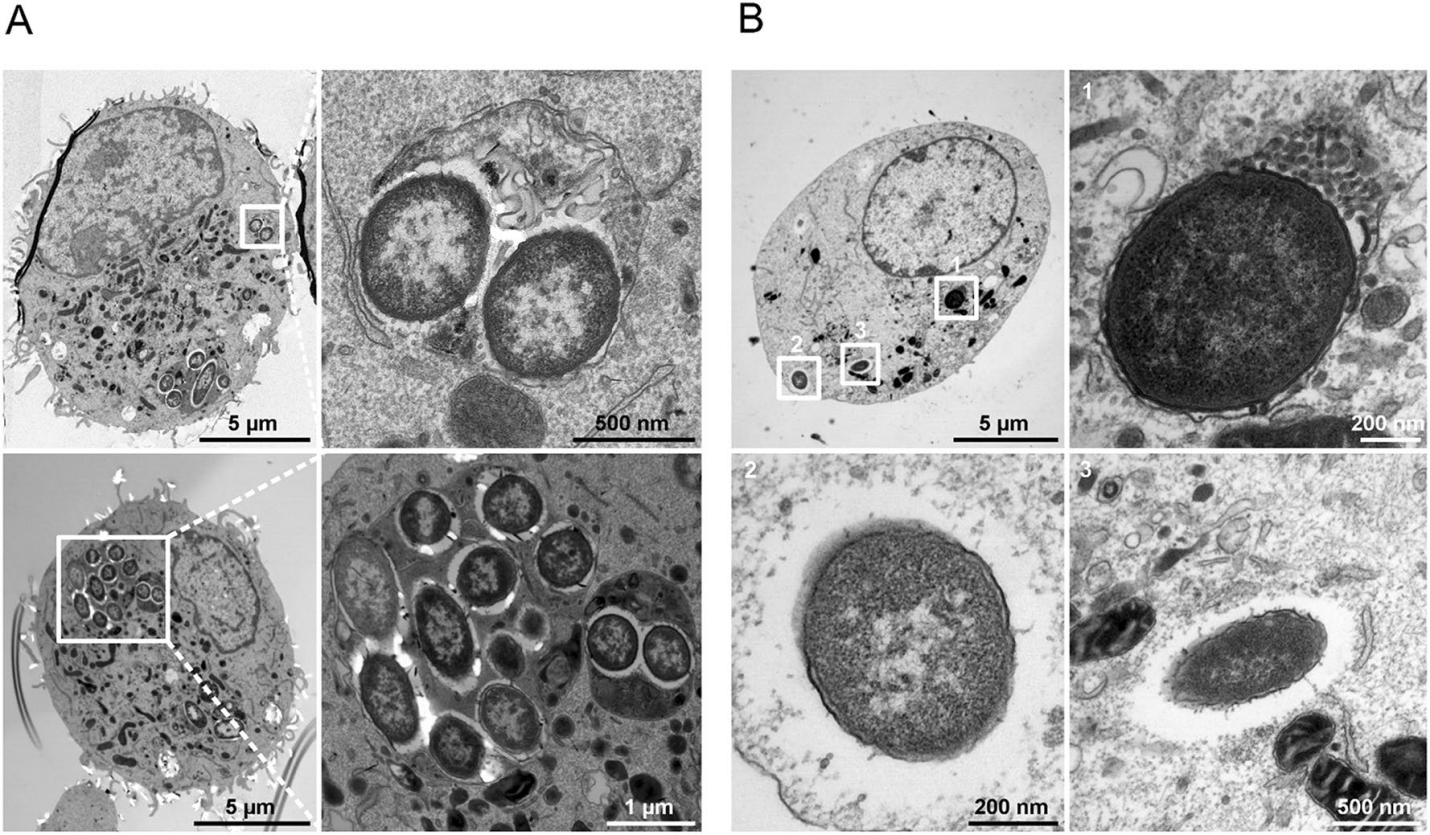
TEM analysis of cultured macrophages infected with *mgtC* mutant strain. J774 macrophages were infected with *P. aeruginosa mgtC* mutant for 2.5 h before fixation for TEM analysis. Phagocytized bacteria reside in healthy macrophages, either inside a vacuole (**A**), which may be a phagolysosome (**B**, panel 1), or in the cytoplasm (**B**, panels 2 and 3).

**Figure 8:**
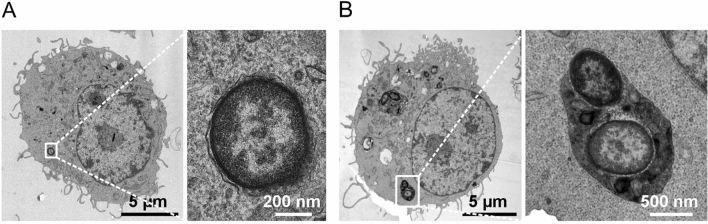
TEM analysis of cultured macrophages infected with *oprF* mutant strain. J774 macrophages were infected with *P. aeruginosa oprF* mutant for 2.5 h before fixation for TEM analysis. Rare phagocytized bacteria reside in healthy macrophages, either inside a vacuole (**A**), or more commonly inside a phagolysosome (**B**).

A similar TEM analysis was conducted with *P. aeruginosa mgtC* and *oprF* mutants. In contrast to the wild-type strain, macrophage lysis driven by intracellular bacteria was not observed with the *mgtC* or *oprF* mutants, which is consistent with our previous results using fluorescence microscopy^16^. In addition, we previously quantified higher bacterial number per macrophage with the wild-type strain compared to the *mgtC* mutant 2.5 h post-phagocytosis and CFU counting confirmed a higher rate of viable surviving bacteria with the wild-type strain^18^. Interestingly, *mgtC* mutant bacteria can be found in macrophages, sometimes with several bacteria residing in a single vacuole (Fig. 7A). In other cases, isolated bacteria could also be found in the cytoplasm or associated with dense material (Fig. 7B), suggesting a fusion of the vacuole with lysosomes. For macrophages infected with the *oprF* mutant, it was more difficult to find evidence of surviving intracellular bacteria, and occasionally, isolated bacteria were found in vacuoles (Fig. 8A) where they were frequently associated with dense material (Fig. 8B), thus also supporting elimination of *oprF* mutant bacteria upon fusion with lysosomes.

In conclusion, TEM analysis showed that the wild-type strain exhibited a lytic phenotype, which was not found with *mgtC* or *oprF* mutant strains, in addition to intracellular survival for at least 2.5 h. Importantly, TEM analysis confirmed a different pattern between *mgtC* and *oprF* mutants in cultured macrophages: *mgtC* mutant could be found in vacuoles at 2.5 h, while at this time point the *oprF* mutant was mostly located in phagolysosomes or already eliminated.

## Discussion

There is an increasing interest in the zebrafish embryo model to monitor *P. aeruginosa* pathogenesis^11^. Here, we further confirmed the pertinence of this model by performing for the first time a comparative analysis of three *P. aeruginosa* strains (PAO1, and *mgtC* and *oprF* mutants) in both mouse and zebrafish models of acute infection. Similar virulence patterns were observed in both models, with an attenuated phenotype for both mutants, and the strongest attenuation for the *oprF* mutant. We then investigated the fate of the three different strains in zebrafish larvae that did not develop an acute fatal infection after 2 dpi, a feature not considered in earlier studies, and demonstrated the relevance of this non-mammalian vertebrate model for tracking *P. aeruginosa* persistence.

Injection of *P. aeruginosa* in closed compartments of zebrafish embryos has been used by several groups to visualize bacteria and their interaction with host innate immune cells^12,28–30^. These studies were done during the acute phase of infection (within 30 hpi). Localized microinjection of zebrafish embryos with *P. aeruginosa* PAO1 and the *oprF* mutant allowed real-time visualisation of macrophages migrating to the injection site to phagocytize bacteria at early time points after infection^12^. In the present study, we conducted real time microscopy analysis of embryos locally injected in the HBV, combined with CFU counting, over a longer time period (up to 4 dpi). Our results indicated that PAO1 has the ability to persist at low bacterial numbers in some larvae that overcome or do not develop acute infection. Injection in the HBV, which is a small compartment, may restrict the number of persisting *P. aeruginosa* and it will be of interest to investigate *P. aeruginosa* persistence using a different infection mode. Real time imaging revealed persisting individual bacteria and bacteria in small clusters, either within or outside macrophages. This finding is consistent with a previous study that visualized bacterial clusters, referred as microcolonies, of *P. aeruginosa* PAO1 at 1 dpi in embryos injected in HBV^28^. In this former study, the analysis was carried out on fixed embryos subjected to immunofluorescence imaging and the intracellular location of bacteria was not investigated. Microcolonies were of smaller size with a *psl* mutant, making the authors suggest that bacterial aggregates may have features of biofilms^28^, although this hypothesis remains to be experimentally validated by other approaches.

**Figure 9:**
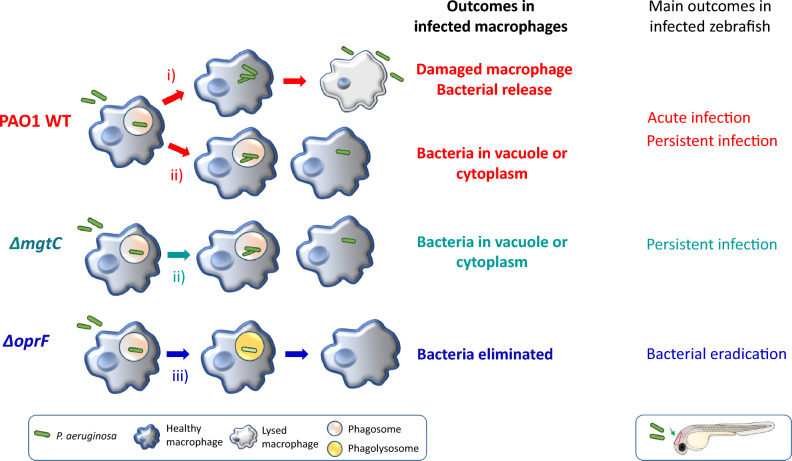
Model for a link between the intramacrophagic fate of *P. aeruginosa* and its ability to persist in zebrafish. TEM analysis of infected cultured macrophages revealed various outcomes including: (i) bacterial release from lysed macrophages, (ii) remaining bacteria in the cytoplasm or in a vacuole in healthy macrophage, (iii) bacterial elimination or bacteria in phagolysosomes. The *P. aeruginosa* PAO1 WT, PAO1Δ*mgtC* and PAO1Δ*oprF* strains differed in their abilities to drive these outcomes in cultured cells, and they also differed in their abilities to drive outcomes in vivo in zebrafish (acute infection, persistent infection or bacterial eradication). By correlating findings in cultured macrophages with findings in zebrafish embryos, we propose that persistent infection could be related to the ability of bacteria to resist killing by macrophages and reside, at least transiently, inside macrophages.

Despite its attenuated phenotype, the *mgtC* mutant also exhibited the capacity to persist at low bacterial numbers, with characteristics similar to those of the wild-type PAO1 strain, namely the ability to reside within macrophages with visible small bacterial clusters up to 3 dpi. On the other hand, the attenuation of the *oprF* mutant was associated with a clear elimination of most bacteria 1 day after infection. Interestingly, the elimination of the *oprF* mutant is consistent with the TEM analysis of infected cultured macrophages, in which the *oprF* mutant was also largely eliminated or found as single bacteria in phagolysosomes at 2.5 h. This finding is in agreement with the higher incidence of localization of the *oprF* mutant with acidified compartments 2 h after phagocytosis^12^. In contrast, TEM analysis of cultured macrophages infected with the *mgtC* mutant showed internalized bacteria, often localized in vacuoles, residing in healthy macrophages at 2.5 h. This is consistent with our previous findings that implicated MgtC in the ability of the bacteria to escape the phagosome^16^. The TEM analysis of wild-type bacteria in infected cultured macrophages showed a more complex picture. First, in contrast to the mutant strains, but in agreement with our previous quantitative analysis using fluorescent microscopy^16^, internalized wild-type bacteria can induce lysis of macrophages and are then released extracellularly. Second, intracellular wild-type bacteria can reside in damaged or healthy macrophages, in vacuoles or in the cytoplasm. Interestingly, vesicles issued from macrophages can harbor *P. aeruginosa*, as observed by TEM and in vivo with the PAO1 strain. Further studies will be required to better characterize these vesicles. Taken together, in vivo infection combined with a cellular infection model with three different strains allowed us to propose a mechanism that links bacterial factors, intramacrophage fate and the outcome of infection (acute infection, persistent infection or bacterial eradication) (Fig. 9). OprF is essential for both acute and persistent infection by *P. aeruginosa*. In contrast, MgtC is required for acute infection in zebrafish larvae and macrophage lysis in cell culture, yet does not seem to be essential for persistent infection in vivo. Of note, the intramacrophage fate of *mgtC* mutant imaged by TEM differs from the one of wild-type PAO1 and further studies are required to fully determine the contribution of MgtC during intramacrophage stages and prolonged infection.

Zebrafish larvae have also been helpful to distinguish *Burkholderia cepacia* complex strains that cause either persistent or acute fatal infection, and to establish a key role for macrophages in bacterial survival and replication during acute infection^31,32^. The importance of an intracellular phase for extracellular respiratory tract bacterial pathogens has gained interest over the past years^33^. Besides *P. aeruginos*a, *Staphylococcus aureus* is another bacterial pathogen classically considered as an extracellular pathogen of the respiratory airway where the intracellular survival and proliferation has been well documented in recent years^33^. Here, our TEM analysis suggests that bacterial persistence may be associated with vacuolar location of the bacteria, at least transiently. This result find echoes in a recent study that identified a subpopulation of *P. aeruginosa* in vacuoles of epithelial cells, which displays persistence features as slow growth and expression of biofilm-associated factors^34^. In our analysis, we only considered the interaction of infecting bacteria with macrophages, but internalization in non-phagocytic cells is likely to also play a role during *P. aeruginosa* long-term infection in animal models. Further studies are needed to explore the localization of intracellular *P. aeruginosa* in the zebrafish embryo model, as well as complementary analysis in an appropriate mammalian model^34^, to better characterize the contribution of intracellular life during chronic infection.

## Data Availability

The datasets used and/or analysed during the current study available from the corresponding author on reasonable request.
